# 3D genome perspective on cell fate determination, organ regeneration, and diseases

**DOI:** 10.1111/cpr.13482

**Published:** 2023-05-17

**Authors:** Hongxin Zhong, Jie Zhang, Yuli Lu, Gongcheng Hu, Guangjin Pan, Hongjie Yao

**Affiliations:** ^1^ CAS Key Laboratory of Regenerative Biology, Guangdong Provincial Key Laboratory of Stem Cell and Regenerative Medicine Guangzhou Institutes of Biomedicine and Health, Chinese Academy of Sciences Guangzhou China; ^2^ GIBH‐CUHK Joint Research Laboratory on Stem Cell and Regenerative Medicine Guangzhou Institutes of Biomedicine and Health, Chinese Academy of Sciences Guangzhou China; ^3^ University of Chinese Academy of Sciences Beijing China; ^4^ Institute of Stem Cell and Regeneration Chinese Academy of Sciences Beijing China; ^5^ Guangzhou Laboratory Guangzhou China

## Abstract

The nucleosome is the fundamental subunit of chromatin. Nucleosome structures are formed by the combination of histone octamers and genomic DNA. Through a systematic and precise process of folding and compression, these structures form a 30‐nm chromatin fibre that is further organized within the nucleus in a hierarchical manner, known as the 3D genome. Understanding the intricacies of chromatin structure and the regulatory mode governing chromatin interactions is essential for unravelling the complexities of cellular architecture and function, particularly in relation to cell fate determination, regeneration, and the development of diseases. Here, we provide a general overview of the hierarchical structure of chromatin as well as of the evolution of chromatin conformation capture techniques. We also discuss the dynamic regulatory changes in higher‐order chromatin structure that occur during stem cell lineage differentiation and somatic cell reprogramming, potential regulatory insights at the chromatin level in organ regeneration, and aberrant chromatin regulation in diseases.

## Graphical abstract

The chromatin is folded and assembled into complex hierarchical structures. Precision control of multiple levels of 3D genome structures (topologically associating domains, chromatin loops, and nucleosomes) is necessary for transcriptional regulation. Cell fate transitions (such as stem cell lineage differentiation and somatic cell reprogramming), organ regeneration, and disease occurrence are all linked to multidimensional chromatin regulation.

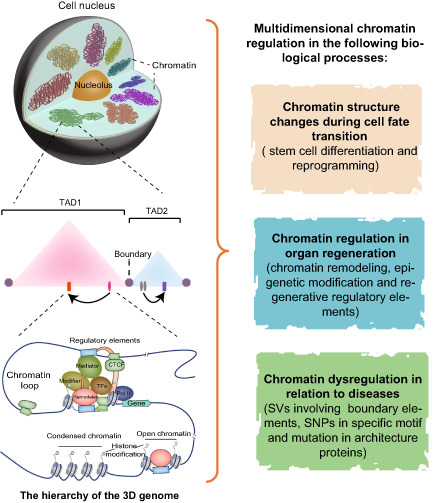

## 1 INTRODUCTION

If stretched end‐to‐end, the diploid human genome is ~2 m long, yet it fits inside a nucleus that is only a few microns across. The chromatin is folded and assembled into a complex hierarchical structure.^1^, ^2^, ^3^ Transcriptional regulation relies on precise 3D genomic architecture.^4^, ^5^ To capture chromatin structure, numerous chromosome conformation capture (3C) techniques and their derivatives, followed by high‐throughput sequencing have been developed in the past two decades.^6^, ^7^ A fresh perspective on the 3D hierarchical organization of the genome has emerged from the analysis of genome‐wide chromatin contact maps, and this has revolutionized our knowledge of chromatin organization as well as our understanding of the transcriptional control of gene expression. Chromatin architectural proteins, such as CCCTC binding factor (CTCF) and the cohesin complex, play the essential roles in the formation of 3D genome.^4^, ^8^ Cell fate changes dynamically during normal development, cell lineage differentiation, organ injury and regeneration, and disease progression. Moreover, cell fate determination is frequently regulated by transcription factors (TFs), chromatin accessibility, and modifications, as well as higher‐order chromatin structure.^9^, ^10^, ^11^ An investigation of multidimensional chromatin architecture provides theoretical guidance for understanding the basic organization of life activities, the regeneration of organs, and the occurrence and development of diseases.

In this review, we present a summary of the hierarchical organization of the 3D genome, discuss the development of 3C techniques and their derivatives in studying the 3D genome, introduce recent discoveries about higher‐order chromatin structure during stem cell lineage differentiation and somatic reprogramming, review the role of chromatin regulation in organ regeneration and diseases, and provide new insights and perspectives for future investigation of these topics.

## 2 HIERARCHICAL STRUCTURE OF THE 3D GENOME

**FIGURE 1:**
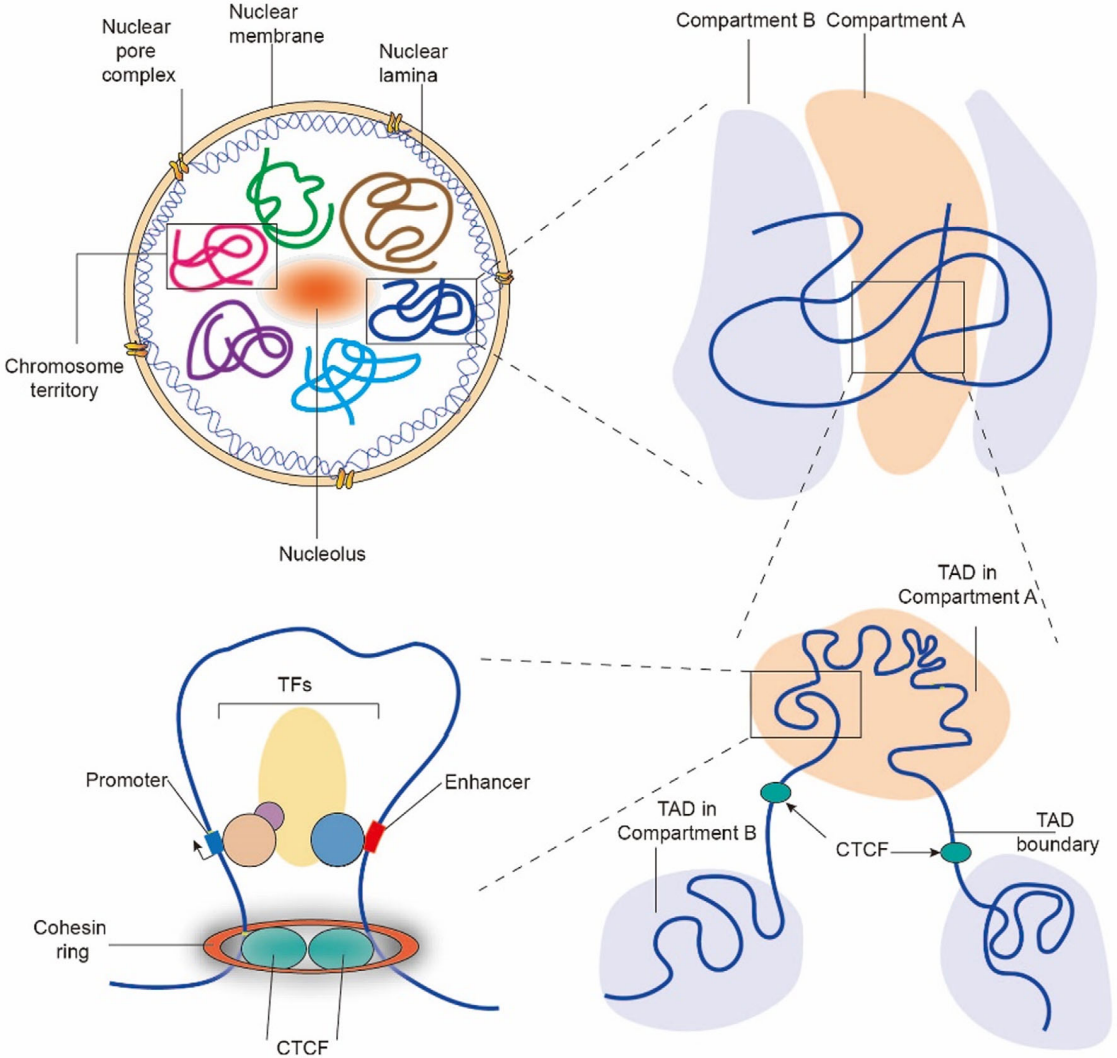
The hierarchy of the 3D genome. During interphase, each chromosome is located at a specific nuclear region, known as chromosome territory. Chromatin can be further divided into active A compartments and relatively inactive B compartments. At the sub‐megabase scale, topologically associated domains (TADs) of local chromatin are maintained by architectural proteins such as CTCF, cohesin complex. According to the loop extrusion paradigm, chromatin loop structures, whose formation is mainly mediated by CTCF and cohesin complex, are enriched in TADs. Chromatin loops can also be maintained by the specific transcription factors.

Functional elements in eukaryotic genomes, including coding genes, non‐coding genes, *cis*‐regulatory elements, repetitive elements, and others, interact specifically in dynamic higher‐order structures rather than being arranged in linear order in the spatial organization. The ‘3D genome’, normally refers to the higher‐order chromatin structure, affects numerous biological processes, including gene expression, DNA replication, DNA damage repair, cell differentiation, and embryonic development.^5^, ^10^, ^12^, ^13^ The multidimensional genome organization of eukaryotes can be described in the following order, from large to small: chromosome territories (CTs), chromatin compartments, topologically associating domains (TADs), chromatin loops (Figure 1).^14^ These structures are associated with gene expression in multiple dimensions and thereby influence the progression of cell fate transitions.

### 2.1 Chromosome territories

During interphase, chromatin could occupy distinct regions within the nucleus known as CTs (Figure 1).^15^ It has been reported that the nuclear topological arrangement of CTs has remained stable over 30 million years of primate evolution.^16^ Several factors contribute to the creation of CTs, including chromosome size and the number of genes present on specific chromosomes. Gene‐dense CTs are enriched inside the nucleus, whereas gene‐poor CTs are located at the periphery of the nuclear membrane.^17^ The spatial arrangement of CTs determines the frequency of genomic translocations. The presence of correct CTs can shield the genome from potentially harmful translocations in the event of DNA damage.^18^

### 2.2 Chromatin compartments

The genome is further separated into active A compartments and inactive B compartments (Figure 1).^19^ The A compartments, which are mainly located in the interior of the nucleus, have the relatively open chromatin structure in contrast to the B compartments, are enriched with housekeeping genes, short interspersed repetitive elements (SINEs), and histone markers such as H3K27ac and H3K36me3 that indicate active transcription. However, the B compartments are primarily located at the edge of the nuclear membrane or around the nucleolus and are associated with tissue‐specific genes, long terminal repeats, long interspersed nuclear elements, and repressive histone modifications such as H3K9me3 and H3K27me3 that are involved in transcriptional silencing.^20^, ^21^ Nuclear re‐localization and A/B compartment switching are regulated by chromatin‐associated proteins such as TFs and chromatin‐modifying enzymes, as indicated by functional analysis of specific genomic loci.^22^, ^23^ The A/B compartments are spatially polarized structures that may contribute to the local concentration of transcriptional machinery and epigenetic regulators and thereby increase the efficient utilization of these biological resources.^24^

### 2.3 Topologically associating domains

Genomic compartments can be further subdivided into topologically associated domains (TADs) at the sub‐megabase scale (Figure 1).^25^ TADs are separated by distinct boundaries and are characterized by the presence of frequent internal chromatin contacts in the same TAD but infrequent interactions across TADs. This means that TADs frequently function as autonomous domains of gene control.^26^, ^27^, ^28^ CTCF, cohesin complexes, SINE elements, tRNAs, housekeeping genes, and active histone modifications such as H3K4me3 and H3K36me3 are abundant at TAD boundaries. These elements are typically closely related to transcriptional activity and epigenetic chromatin modification signatures.^26^ As an insulator at the boundary, CTCF prevents the interaction of regulatory elements located on two nearby TADs.^29^ Furthermore, TADs can be further divided into smaller sub‐topological domains (sub‐TADs) that are highly conserved across species and stable across diverse cell types.^27^ TADs are functional units of chromatin, and co‐regulated genes located in a given TAD exhibit comparable patterns of expression during differentiation.^25^, ^30^ Disruption of TAD boundaries could result in aberrant chromatin interactions, long‐range transcriptional dysregulation, altered gene expression, and diseases.^25^, ^31^

### 2.4 Chromatin loops

At the kilobase scale, chromatin fibres condense into loop‐like structures known as chromatin loops (Figure 1).^32^ The ‘loop extrusion model’ is currently one of the most well‐known models that explain how chromatin loops are formed. The cohesin complex movement is halted when it encounters CTCF‐occupied sites while sliding and compressing along chromatin.^33^ The chromatin loops mediated by CTCF and the cohesin complex within a specific chromatin environment are intimately associated with gene expression.^34^ Direct evidence for the loop extrusion paradigm has been provided by in vitro single‐molecule imaging technology showing that the cohesin‐NIPBL complex may compress DNA through ATP‐driven loop extrusion.^35^ At a finer scale, the cohesin‐associated loop extrusion machinery can be further helped by RNA polymerase II when the transcriptional machinery and cohesin complex clash, which may cause the development of enhancer–promoter loop or promoter–promoter loop.^36^

Chromatin loops is closely related with regulation of gene expression.^37^ Our recent study revealed that CTCF has an alternative splicing isoform that skips Exons 3 and 4, causing translation to start at Exon 5 and resulting in a shortened CTCF protein (CTCF‐s).^38^ Competition between CTCF‐s and classical CTCF for DNA binding results in disruption of the CTCF‐mediated chromatin loops and promotes apoptosis through activation of interferon‐inducible protein 6 (IFI6). CTCF, along with other potential factors, governs long‐range chromatin interactions. We screened the factors that may be involved in the regulation of chromatin loop formation and indicated that basic helix–loop–helix family member e40 (BHLHE40) regulates CTCF binding and thereby impacts the stability of chromatin loops.^39^ Additionally, Yin Yang 1 (YY1) functions as a chromatin structural protein by mediating the interaction between enhancers and promoters and thus regulates gene expression.^40^ However, current investigations have shown that acute depletion of architectural proteins (CTCF, cohesion, WAPL, and YY1) using a degradation system had relatively moderate effects on the expression of most genes though these proteins mediate chromatin loops,^41^, ^42^ indicating that the dynamic changes of higher‐order chromatin structure are not positive correlation with the gene expression. Therefore, the relationships between higher‐order chromatin structure and gene expression need us to further investigate.

## 3 CHROMOSOME CONFORMATION CAPTURE AND ITS‐ASSOCIATED TECHNOLOGIES

### 3.1 The development of 3C method and its derivatives

**FIGURE 2:**
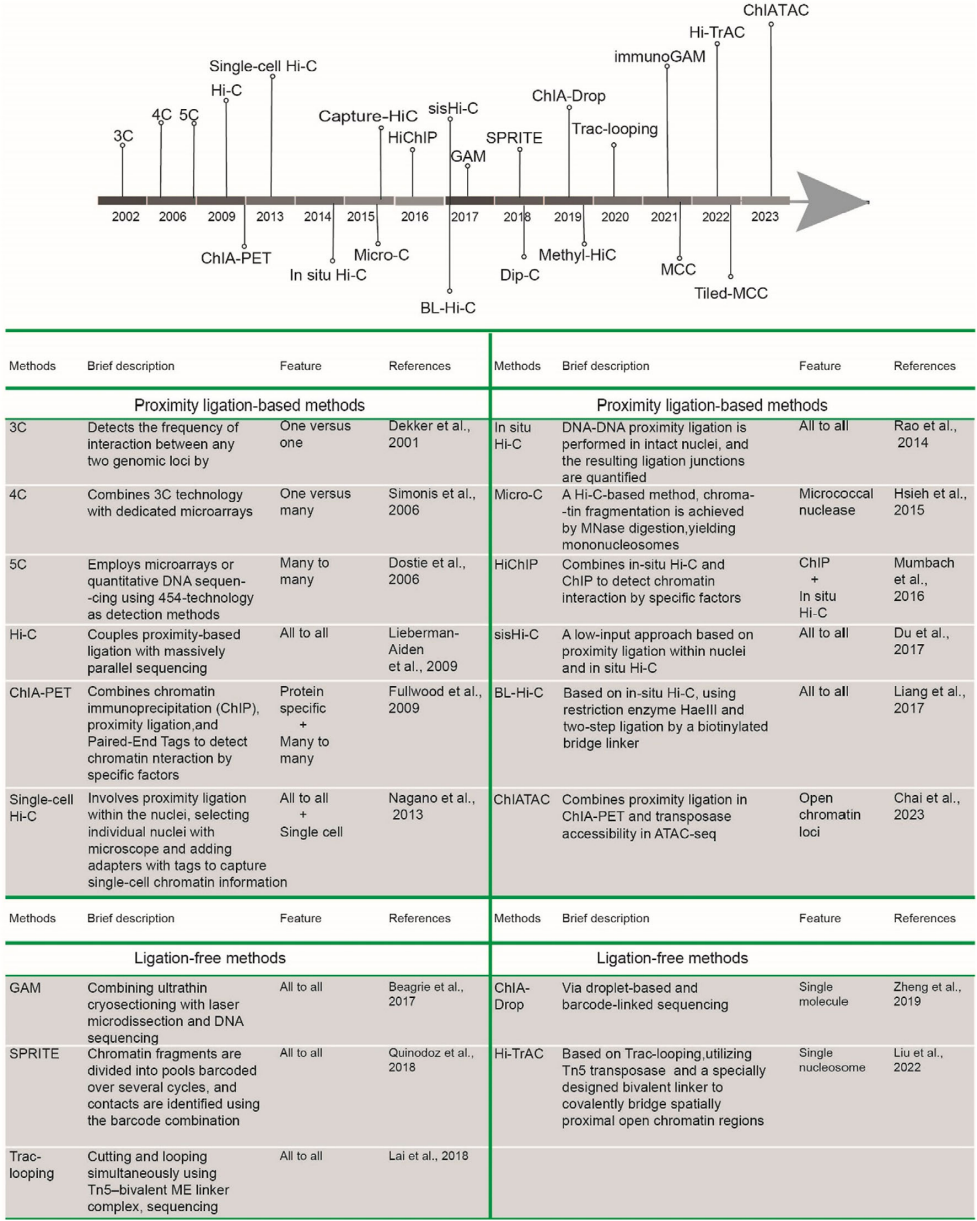
A timeline and table depicting the development of crucial chromosome conformation capture and the derived technologies.

The spatial conformation of chromatin is one of the essential components of transcriptional regulation. Advances in 3C and 3C‐based derivative technologies over the past two decades have made it easier for us to study 3D genome organization (Figure 2).^19^, ^43^ 3C was initially reported in 2002, which could be used to deduce the spatial architecture of chromatin based on quantitative measurement of the frequency of interactions between two loci.^43^ Based on the 3C method, circular chromosome conformation capture or chromosome conformation capture‐on‐chip  (4C) technology for capturing all loci that interact with a single specific locus,^44^, ^45^ and chromatin conformation capture carbon copy (5C) technology used for detecting all interactions within a specific locus,^46^ were rapidly developed. Hi‐C technology, which makes genome‐wide chromatin interaction analysis possible, was developed in 2009.^19^ Despite the shortcomings of the initial version of the Hi‐C technique, including high expenses, complicated and time‐consuming experimental methods, lots of randomly ligated DNA noise, and a lack of simple noise evaluation, Hi‐C marks the beginning of the era of mapping genome‐wide chromatin interactions.

### 3.2 Derivative techniques based on Hi‐C

The most notable improvement of the Hi‐C technique is the invention of in situ Hi‐C technology.^32^ In situ Hi‐C employs a specific four‐base cutter enzyme to fragment DNA, enhancing the number and diversity of DNA fragments and boosting sequencing coverage and resolution relative to Hi‐C using a six‐base cutter enzyme. Furthermore, in situ Hi‐C executes adjacent ligation and previous steps in the nucleus, thereby capturing chromatin interactions closer to its natural state. In situ Hi‐C has overcome the shortcomings of traditional Hi‐C, and has been widely adopted and improved upon in subsequent versions. For example, sisHi‐C,^47^ an assay that captures information from a small number of cells by optimizing the procedure based on in situ Hi‐C, was used for mapping chromatin interactions of early embryonic samples. Bridge‐Linker Hi‐C, an improved method in which biotinylated linker sequences are used, makes the technique more efficient and sensitive in capturing active chromatin interactions.^48^ Micro‐C significantly improves the resolution of the Hi‐C method by using micrococcal nuclease (MNase) to cleave the genome.^49^

In addition to the methods described above for capturing whole genome chromatin interactions, the techniques for capturing local chromatin interactions are being developed. Micro‐Capture‐C (MCC)^50^ and Tiled‐MCC^51^ technologies, both of which are based on Micro‐C, were developed for high‐resolution chromatin interaction of specific loci. As needed, other derivative technologies centred on specific factors (e.g., TFs or histones) or specific elements (e.g., promoter or enhancer regions) were established, such as ChIA‐PET,^52^ HiChIP,^53^ Capture Hi‐C,^54^ ChIATAC,^55^ and others (Figure 2). Furthermore, the development of single‐cell Hi‐C technology allowed us to detect thousands of chromatin contacts in single cells.^56^ In addition, the optimized versions of single‐cell Dip‐C^57^ and Methyl‐HiC^58^ successively emerged after that.

### 3.3 Other ligation‐free methods

Simultaneously, nonadjacent‐junction approaches have been developed to avoid the inherent linkage bias of standard adjacent‐junction methods; these include genome architecture mapping (GAM)^59^ and its derived immunoGAM,^60^ SPRITE^61^ and ChIA‐Drop^62^ (Figure 2). These technologies have advantages in capturing long‐range and multiway contacts at TAD scale and have been described in detail.^63^ Other ligation‐free methods such as Trac‐looping^64^ and its derived high‐resolution technology, Hi‐TrAC^65^ exploit the bridging ability of TN5 transposase to reveal fine and comprehensive interactions between regulatory elements at the chromatin loop level.

## 4 3D GENOME IN CELL FATE DETERMINATION

The advances in 3D genome technology have linked the regulation of chromatin hierarchy to cell fate transitions. A number of reviews have described the contribution of the 3D genome to early embryonic development, cell lineage differentiation, and cell fate transitions.^9^, ^10^ Here, we mainly focus on the role of CTCF‐mediated chromatin regulations in cell fate determination.

### 4.1 Chromatin structure and stem cell differentiation

Embryonic stem cells can generally differentiate into intermediate‐state progenitor cells of different lineages and thence into terminally differentiated somatic cells according to established routes.^66^ Chromatin compartments experience dynamic changes as stem cells transition to other cell types during differentiation. For instance, 36% of A and B compartments in the genome switches during the differentiation of human embryonic stem cells (hESCs). However, the observed changes in gene expression are subtle, which suggests a little link between switches in compartments and changes in gene expression during hESC differentiation.^67^ TAD borders have been shown to form early in development and remain stable during differentiation. In contrast, the differentiating cells undergo a significant reorganization of the contacts inside TADs, resulting in the creation of particular interactions between enhancers and promoters. Studies of the differentiation of mouse embryonic stem cells (mESCs) into neural progenitor cells (NPCs) also revealed significant structural reorganization of the genome on the sub‐megabase scale.^27^ Loss of stem cell pluripotency is accompanied by the rearrangement of chromatin loops. CTCF, cohesin complex, and YY1, which are especially abundant in NPC‐specific chromatin loops, control how chromatin loops interact at different scales.^27^, ^68^, ^69^ V(D)J rearrangement is a key event in the development and maturation of immune cells.^70^ The dynamic process of chromatin loop extrusion driven by CTCF is an important mechanism of V(D)J rearrangement and the generation of antibody diversity in immune cells.

As mentioned earlier, CTCF usually acts as an insulator at the TAD boundary and prevents the interactions between the two sides of the regulatory elements. Simultaneously, CTCF also mediates loop extrusion together with the cohesin complex, which regulates the mutual interaction of distant regulatory elements. Although acute degradation of CTCF in cell lines has little effect on gene expression, prolonged degradation of CTCF leads to morphological abnormalities and cellular differentiation.^41^ In mESCs, CTCF degradation promotes the spontaneous and asynchronous transformation of cells into 2C‐like cells, and this effect is reversible when CTCF levels are restored.^71^ This 2C‐like transition is uniquely observed in pluripotent cells in response to CTCF degradation. Similarly, degradation of CTCF in mESCs during neural differentiation delays axonogenesis, and CTCF binding at cell type‐specific gene promoters drive CTCF‐dependent EP interactions.^72^ CTCF is also necessary for the differentiation of mESCs into myocardial mesoderm and mutation of lysine 20 in CTCF suppresses mESC differentiation into early cardiac mesoderm by reducing CTCF‐mediated chromatin binding and chromatin interactions, as well as chromatin accessibility.^73^ It was recently discovered that CTCF also controls the higher‐dimensional structure of chromatin at the level of compartments. Specifically, CTCF controls the interaction of inter‐A compartments in the form of phase separation via RYBP (RING1 and YY1 Binding Protein), thereby preventing the differentiation of mESCs into neural progenitors.^74^

The critical function of the 3D genome in deciding cell fate transition has been enriched by studies of how chromatin structure is mediated by architectural proteins during cell lineage differentiation. Further investigation is needed to allow us to understand the relationships between architectural proteins and lineage‐specific specification, how these proteins coordinate in the lineage differentiation process, and whether there are distinct regulatory paradigms in chromatin organization during the differentiation of distinct lineages.

### 4.2 Chromatin structure and reprogramming

Terminally differentiated cells undergo cell fate reversal through somatic cell nuclear transfer (SCNT), overexpression of TF sets, or induction by small molecule compounds.^75^, ^76^, ^77^ Conversion of somatic cells to induced pluripotent stem cells (iPSCs) could be executed by either TFs or small molecules.^76^, ^77^, ^78^ During transdifferentiation (also known as direct reprogramming), the cells switch directly from one lineage to another without going through a pluripotent state. SCNT technology transforms cells into a totipotent form similar to the cells present in zygotes. Here, we further review how chromatin structure participates in the reprogramming of somatic cells into iPSCs.

Since Takahashi and Yamanaka^76^ successfully transformed mouse embryonic fibroblasts (MEFs) into iPSCs using four TFs, OSKM (Oct4, Klf4, Sox2, and c‐Myc). This system for cell pluripotency acquisition has been widely used to investigate the biological mechanisms that underlie cell fate transition. Somatic cell reprogramming involves the suppression of somatic gene expression programmes and the activation of pluripotency programmes. During reprogramming, chromatin is dynamically reorganized, and multiple genetic and epigenetic barriers must be overcome. Previous studies used multi‐omics techniques to depict the rearrangement of TFs in enhancer regions and the remodelling of the epigenetic landscape during reprogramming.^79^, ^80^ Enhancer elements are essential for cell type‐specific gene expression as TF binding platforms. During the reprogramming process, TFs such as OCT4, SOX2, and KLF4 slowly shift from early enrichment at somatic cell‐associated enhancers to late enrichment at pluripotency enhancers, thereby altering the gene expression programme.^81^, ^82^ After effectively eliminating the tissue‐specific 3D genome structure, iPSCs derived from multiple lineage cells are able to establish an embryonic stem cell‐like 3D genome.^83^ A recent critical study revealed that the overall changes in gene expression, genome topology, and chromatin state are tightly related.^84^ But in general, this relationship occurs non‐synchronously: changes in transcription often come after changes in sub‐nuclear compartmentalization, TAD connectivity, and the strength of TAD‐border insulation. During cell reprogramming, TFs cause a series of effects to the genome structure and chromatin state, which permits the rewiring of the gene regulatory network.

The interactions between long‐distance enhancers and promoters are crucial in regulating chromatin structure and gene expression. For instance, removing individual KLF4 binding sites inside pluripotent stem cell‐specific enhancers was sufficient to damage EP interactions and decrease the expression of related genes during the reprogramming of MEFs into iPSCs.^85^ During the reprogramming of mouse pre‐B cells to pluripotent cells, reorganization of chromatin compartments and TAD structures, as well as alterations in cell type‐specific chromatin loops, occur in a stage‐specific manner.^84^ The reorganization of chromatin during somatic reprogramming is also associated with TF‐driven phase separation.^86^ For example, OCT4 modulates TAD reprogramming by altering CTCF binding at the TAD boundary through phase separation. CTCF plays a vital role in 3D genome organization during the reprogramming process. Moreover, we recently discovered that CTCF represses the expression of somatic genes by acting as a chromatin insulator and functions as a chromatin remodeller to maintain the accessibility of pluripotency genes.^87^

## 5 CHROMATIN STRUCTURE AND ORGAN REGENERATION

Organ regeneration is a major goal of regenerative medicine, which includes the regeneration and repair of damaged tissues as well as the rebuilding of organs using stem cell technology. The essence of regenerative biology at the cellular level is associated with cell fate transition. Therefore, it is crucial to examine how programmes involved in regeneration are affected by changes in the chromatin environment. The regulatory mechanisms that operate at the chromatin level during regeneration events have been extensively studied using model animals such as *Hydra*, *Drosophila*, zebrafish, and mice.^11^ To offer fresh perspectives on chromatin organization during regeneration processes, we mainly focus on the multidimensional regulation of chromatin during regeneration events; this multidimensional regulation involves chromatin remodelling, epigenetic alterations, and regulatory elements that participate in regeneration.

### 5.1 Chromatin remodelling

By changing the physical spacing of the nucleosomes, ATP‐dependent chromatin remodelling complexes (SWI/SNF, ISWI, CHD, and INO80) make DNA more accessible to particular protein factors and thereby change gene expression programmes.^88^ In studies of liver regeneration in mice, SMARCA4, a component of the SWI/SNF remodelling complex, was found to activate the Wnt/β‐catenin pathway by interacting with β‐catenin protein and thereby promote hepatocyte proliferation. Deletion of SMARCA4 inhibits liver regeneration and affects survival after hepatectomy.^89^ In contrast, deletion of the SWI/SNF complex component ARID1A significantly improves organ regeneration after liver injury in mice.^90^ Therefore, the balance of chromatin remodelling complex components may be one of the factors limiting tissue regeneration. The role of chromatin remodelling factors in maintaining differentiation‐related programmes and activating regeneration‐related programmes at the level of chromatin conformation remains to be further studied. SMARCA4 has been shown to bind the enhancers of Myc genes in leukaemia cells and is required for the regulation of distant chromatin interactions.^91^ In human mammary epithelial cells, deletion of SMARCA4 leads to significant changes in higher‐order chromatin structure.^92^ Our recent study showed that SMARCA5, the ATPase of the ISWI complex, cooperates with CTCF to maintain chromatin accessibility and promote the reprogramming of mouse fibroblasts into iPSCs.^87^ The regulatory regions of proliferation‐related genes in regenerating hepatocytes display increased chromatin accessibility and are enriched with ELK1 and CTCF, according to a study of dynamic chromatin architecture in regenerating liver.^93^ Further study should be required to understand how chromatin remodelling and higher dimensional chromatin conformations change during regenerative events and whether these changes are dependent on CTCF and cohesin complex‐mediated chromatin structure. Obtaining this information may open up new avenues to understanding how regenerative programmes are initiated and processed.

### 5.2 Epigenetic modifications

The studies related to 3D genome in regulating regeneration are really rare; however, epigenetic modifications are highly connected to 3D genomic regulation.^94^, ^95^ Epigenetic modifications such as histone modification and DNA methylation are also involved in organ regeneration after injury. Polycomb groups (PcGs) are classical epigenetic inhibitory complexes that are divided into two main types of complexes, PRC1 and PRC2.^96^ These complexes mediate the ubiquitination of histone H2A on lysine 119 (H2AK119ub) catalysed by RING1A/B and the trimethylation of histone on lysine 27 (H3K27me3) catalysed by EZH1/2, respectively.^96^ In regeneration‐related studies, EZH2‐deficient zebrafish failed to regenerate after caudal fin transection injury, suggesting that EZH2 and the histone modifications may play a role in the regeneration process.^97^ In contrast, during cardiac regeneration in mice, EZH1, but not EZH2, is required to activate cardiac regeneration‐related genes,^98^ suggesting that PcG may play a role in both transcriptional repression and transcriptional activation during regeneration events. Several recent studies have also shown that, unlike the classical repressive process, PcG can activate gene expression programmes during cell fate transition.^99^, ^100^, ^101^ Comparison of multi‐omics data from quiescent and regenerating hepatocytes in mouse liver revealed that pro‐regeneration‐related genes tended to be found in the active region of the genome and were enriched in the H3K27me3 modification in the resting state; this modification was erased during regeneration, and proliferation‐associated gene expression programmes were quickly activated.^102^ The multiple regulatory actions of PcG can be explained by the existence of higher dimensional chromatin structural network. Specifically, detailed suggestions regarding the role of PcG in 3D genome folding have been made.^103^ Thus, it may be possible to determine how the PcG complex orchestrates the regeneration programme at the level of chromatin structure in the future.

### 5.3 Regenerative regulatory elements

**FIGURE 3:**
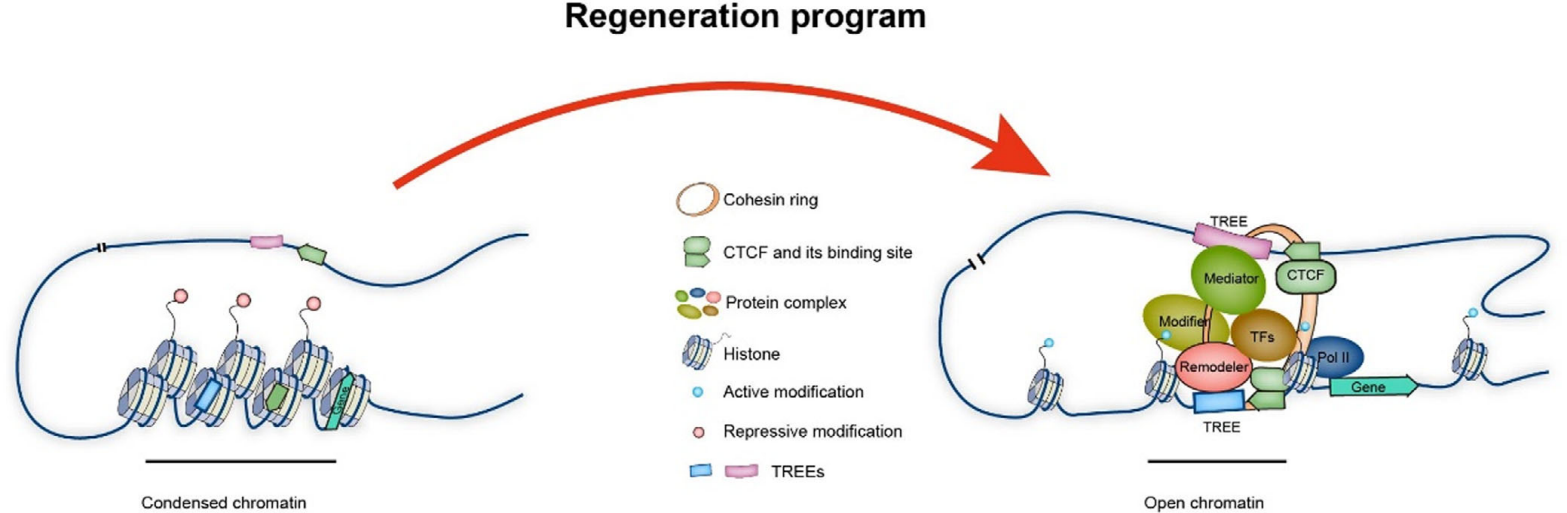
Chromatin conformation during the regeneration programme. The activation of the regeneration‐associated gene expression programme is chromatin context‐dependent. Before initiating the regeneration programme, the tissue regeneration enhancer elements (TREEs), CTCF binding sites, and regeneration‐associated genes are localized at the dense chromatin. When the regenerative programme is initiated, chromatin remodelling complexes facilitate to remodel chromatin, transcription factors, and epigenetic modifiers regulate gene transcription. Moreover, CTCF, cohesin, and mediator complexes orchestrate long‐range interactions of TREEs, resulting in the formation of specific chromatin conformations and chromatin hubs.

Chromatin regulatory elements (e.g., promoters and enhancers) play an essential role in gene expression. With the development of multi‐omics technologies, chromatin regulatory elements associated with regeneration events have been identified in *Hydra*, *Drosophila*, zebrafish, and other organisms by integrating omics information on chromatin state and histone modifications.^104^, ^105^, ^106^ The results show that some elements are relatively conserved among different species. For example, a class of tissue regeneration enhancer elements (TREEs) identified in regenerating tissues of zebrafish can trigger the repair programme at the injury site. TREE can also induce reporter gene expression at injury sites in mice.^106^ Recent studies have used TREE and recombinant adenoviral AAV vectors to achieve precise repair of damaged areas of the mouse heart, demonstrating the great potential of TREE in regenerative medicine applications.^107^ The spatial interaction of chromatin regulatory elements is an important part of gene expression, but the mechanism by which TREEs drive regeneration‐related gene expression remains to be studied. Is the appropriate 3D chromatin structure between TREE and regeneration‐related genes the basis for their function? We propose that a stable chromatin conformation is required for the initiation and maintenance of a regeneration programme and that this can be achieved by the coordinated action of TFs, remodelling complexes, mediators, epigenetic modification, TREE, and architectural proteins (Figure 3). Therefore, further analysis of the multidimensional chromatin environment near TREEs will help us to understand the regulatory process that occurs during regeneration events and further guide clinical application.

## 6 CHROMATIN STRUCTURE AND DISEASES

Recently, a number of studies have taken advantage of 3C‐derived techologies combined with high‐throughput sequencing to study the relationships between disease‐related genetic variation and chromatin conformation. Abnormal regulation of chromatin structure in diseases has become a focus of current research.

### 6.1 Genetic variation linked to abnormal chromatin structure

**FIGURE 4:**
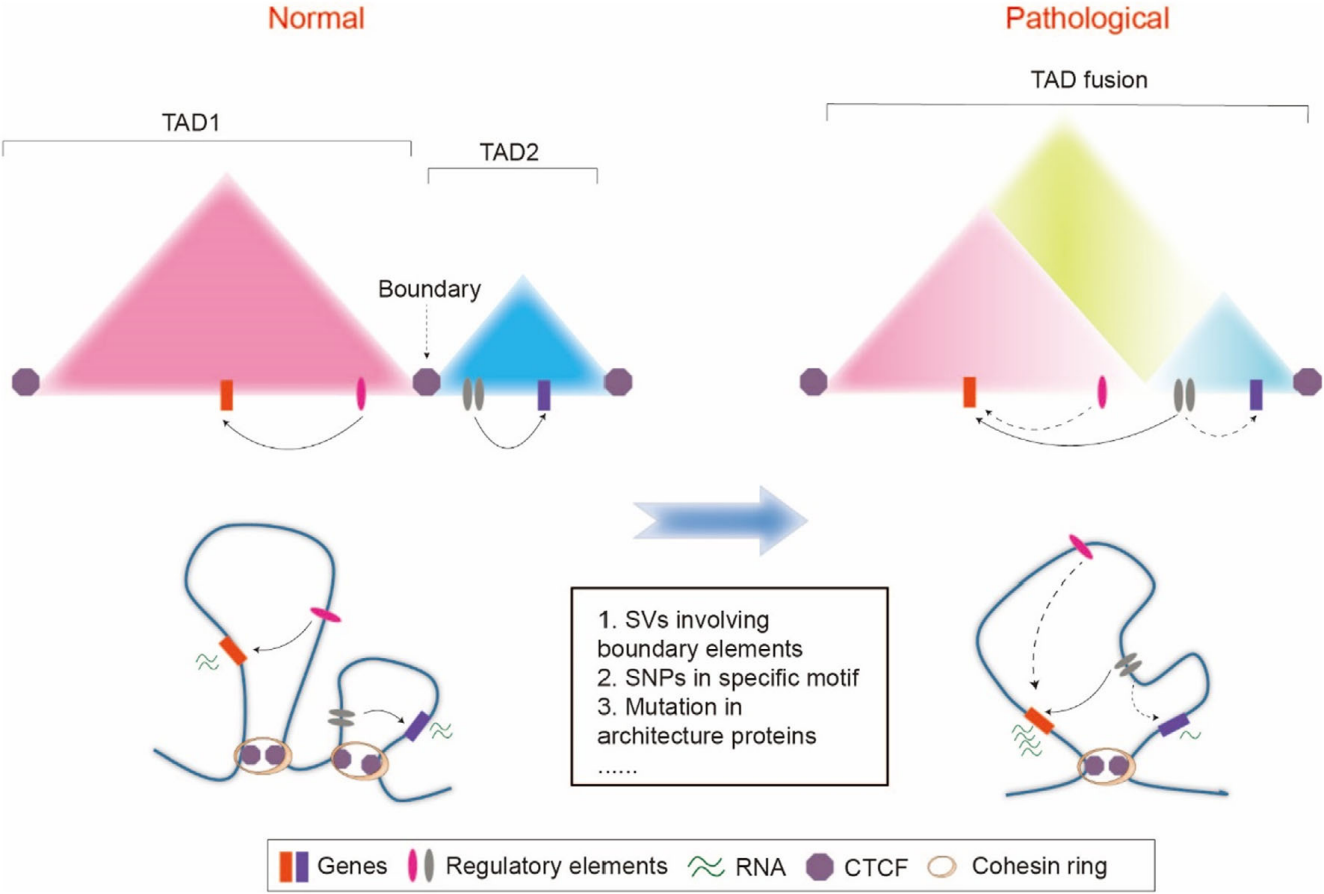
A 3D genomic view of disease dysregulation. Under normal conditions, genes and their regulatory elements are located within specific topologically associating domain (TAD) structures, and the regulatory elements between adjacent TADs do not interfere with each other. However, under the pathological conditions in which the disease‐related structural variants (SVs) involving TAD boundary elements (e.g., the sequence deletion of boundary elements may lead to TAD fusion), the regulatory elements between adjacent TADs begin to interfere with one another, and this can disrupt gene regulation and cause diseases. To a certain extent, single nucleotide polymorphisms (SNPs) in regulatory factor binding motifs and mutations in architectural proteins can also produce aberrant chromatin conformational patterns and pathogenic phenotypes.

Genomic structural variants (SVs; including deletions, duplications, inversions, translocations, and insertions of sequences on the genome) and single nucleotide variants are common causes of genetically inherited developmental diseases and cancer.^108^ To a certain extent, disease‐associated SVs cause variation in the spatial conformation of the genome, and this may be due to aberrant regulation of the higher‐order structure of chromatin, which in turn leads to observable disease phenotypes. For example, genomic structural variation at the TAD boundary may cause rearrangement of EP contacts, resulting in abnormal expression of disease‐related genes. Single nucleotide polymorphisms (SNPs) in non‐coding regions may also contribute to abnormal chromatin structure, usually as a result of SNPs affecting specific TF binding motifs or specific DNA methylation sites, ultimately affecting the overall chromatin conformational environment (Figure 4).^109^, ^110^, ^111^

#### 6.1.1 The examples of developmental diseases

Multiple developmental disorders have been linked to specific loci in chromatin. At the EPHA4‐PAX3 locus, for instance, genomic variation has been associated with several rare limb malformations. A linear distribution of *WNT6*‐*IHH*‐*EPHA4*‐*PAX3* genes in three TADs separated by two boundary elements characterizes this locus, at which the enhancer element and the *EPHA4* gene are in the same TAD. Pathogenic structural variations at these loci in humans include fragment deletions, inversions, and duplications that may cause visible finger malformation phenotypes if the variant involves the boundary element. For example, deletion of *EPHA4* at the TAD boundary results in the fusion of two TADs and aberrant contact of the enhancer with the *PAX3* gene, leading to misexpression of PAX3 and a brachydactyly phenotype. Inversion of the enhancer leads to abnormal activation of *WNT6* and causes syndactyly, while duplication of the enhancer and the *IHH* sequences leads to misexpression of the *IHH* gene, causing polydactyly.^31^ The *EPHA4‐PAX3* locus contains tandem CTCF sites at the TAD boundary, which are conserved in mice. Recent study has used animal models to analyse in great detail how each CTCF at the boundary affects gene expression, enhancer insulation, and pathological traits. Studies have revealed that tandem CTCFs serve redundant roles and that insulation of the boundary can have a direct impact on gene expression and on the accompanying phenotype.^112^ Genomic SVs may also lead to the generation of new TAD structures, resulting in disease‐associated dysregulation of gene expression, primarily due to the duplication of boundary elements. For example, replication of the *IGF2* locus has been observed in colorectal cancer patients and shown to lead to new TAD formation and aberrant overexpression of *IGF2*.^113^ If a duplication of the *SOX9* locus involves the neighbouring *KCNJ2* gene, a new TAD will be created, and the *KCNJ2* gene will be in aberrant contact with the *SOX9* regulatory element and misexpressed, resulting in limb deformity. The generation of aberrant chromatin structures by sequence duplication is also related to copy number and duplication area. If the duplication around the *SOX9* locus does not include the *KCNJ2* gene, it does not cause misexpression or a disease phenotype.^114^

#### 6.1.2 Enhancer hijacking: a critical phenomenon in chromatin aberrations

SVs in genomic DNA have been linked to abnormal chromatin structure, leading to disease‐associated dysregulation of gene expression. However, the production of abnormal regulation of gene expression usually requires certain conditions to be met, such as changes in boundary elements and ectopic chromatin contacts. One of the critical phenomena related to ectopic contacts in chromatin is ‘enhancer hijacking’,^115^ in which SVs lead to aberrant regulation of the expression of disease‐associated genes by enhancers. In several recent tumour‐related studies, it was discovered that enhancer hijacking events are the driving factors of high levels of oncogene expression.^116^ Algorithmic tools have been developed to identify SVs that may cause enhancer hijacking events.^117^ These studies enrich current mechanistic studies on how chromatin structure is related to cancer development.

### 6.2 Mutations of architectural proteins are associated with developmental disorders

There is mounting evidence that connects the occurrence of diseases to anomalies in chromatin arrangement. Mutations in the genes that encode architectural proteins are another form of connection between structural changes in chromatin and developmental disorders (Figure 4). According to the information gained from studies of several recent clinical cases and large‐scale sequencing research on developmental disorders, mutations in genes related to CTCF and cohesin complexes are frequently connected to the occurrence of neurodevelopmental disorders.^118^, ^119^, ^120^, ^121^, ^122^, ^123^, ^124^, ^125^ These genetic mutations, including truncation or functional amino acid mutations, disrupt the normal chromatin biology of these proteins, resulting in more widespread effects compared with those caused by variations in specific sequence element, such as boundary elements, in SVs. Aberrant expression of certain tissue‐specific genes influenced by chromatin structural regulation may be a comprehensive manifestation of developmental disorders featuring multi‐tissue abnormalities, as previous studies have associated certain cell and tissue‐specific chromatin conformations with gene expression and specialized functions.^60^ Chromatin insulation mediated by CTCF and cohesin complexes, as well as chromatin loop dysfunction, might be the main cause of these developmental issues. However, further study is required using relevant mutant cell models or disease animal models to elucidate in detail.

Understanding the occurrence and progression of diseases from the standpoint of chromatin structural variation will thus be a future direction for improving disease diagnosis. On the one hand, more sophisticated detection methods and computational tools are required to make it possible to discover relevant chromatin structural alterations. On the other hand, gene editing approaches could be used to create equivalent cell or animal models to explore the possible regulatory processes. Disease simulation and mechanism studies could benefit from using human iPSC‐derived organoid technology, which has shown great promise in disease modelling and regenerative medicine in recent years.

## 7 SUMMARY AND PROSPECTS

This review summarizes the recent history of 3C and its derived techniques and describes their application and direction for revealing the role of chromatin architecture in stem cell differentiation, somatic cell re‐programming, organ regeneration, and diseases. Examination of the 3D structure of chromatin and the mechanisms that govern chromatin interactions is necessary to gain insight into how cells coordinate their most basic biological functions. Theoretically, understanding how chromatin organization changes dynamically during cell fate transition could help us to create in vitro cell treatment materials that are more effective and safer than currently available. Investigation of chromatin architecture in regeneration and diseases provides theoretical guidelines for in vitro simulation of organ regeneration and disease development with potentially clinical usefulness. However, there are several limitations of the current study: First, the lack of high‐resolution methods to capture 3D chromatin interactions at single cell type or even single cell level. Second, the scarcity of data on 3D genome organization in various cell types, developmental stages, and regeneration processes. Third, the difficulty in correlating and integrating multi‐omics data such as chromatin structure, epigenetic modifications, TF networks in the same biological event, and so forth. Therefore, the utilization of high‐resolution technologies in the future may be necessary to resolve chromatin conformation at the level of single cell types or even single cells. In addition, coordination of multi‐omics sources and functional analysis will be required to comprehensively delineate the rules of chromatin regulation in cell fate transition, regeneration events and disease process.

### AUTHOR CONTRIBUTIONS

Hongxin Zhong, Jie Zhang, and Yuli Lu wrote the first draft of this paper. Gongcheng Hu and Guangjin Pan helped to prepare for this work. Hongjie Yao supervised and approved the final version.

## FUNDING INFORMATION

This work was supported by the Strategic Priority Research Program of the Chinese Academy of Sciences (XDA16010502), National Key R&D Program of China (2021YFA1100300), and National Natural Science Foundation of China (U21A20195, 31925009).

## CONFLICT OF INTEREST STATEMENT

The authors declare that they have no competing interests.

## Data Availability

Data sharing is not applicable to this article as no new data were created or analysed in this study.

## References

[1] Misteli T . Beyond the sequence: cellular organization of genome function. Cell. 2007;128(4):787‐800. 17320514 10.1016/j.cell.2007.01.028

[2] Razin SV , Ulianov SV . Gene functioning and storage within a folded genome. Cell Mol Biol Lett. 2017;22(1):18. 28861108 10.1186/s11658-017-0050-4 PMC5575855

[3] Misteli T . The self‐organizing genome: principles of genome architecture and function. Cell. 2020;183(1):28‐45. 32976797 10.1016/j.cell.2020.09.014 PMC7541718

[4] Merkenschlager M , Nora EP . CTCF and Cohesin in genome folding and transcriptional gene regulation. Annu Rev Genomics Hum Genet. 2016;17:17‐43. 27089971 10.1146/annurev-genom-083115-022339

[5] Xu H , Zhang S , Yi X , Plewczynski D , Li MJ . Exploring 3D chromatin contacts in gene regulation: the evolution of approaches for the identification of functional enhancer‐promoter interaction. Comput Struct Biotechnol J. 2020;18:558‐570. 32226593 10.1016/j.csbj.2020.02.013 PMC7090358

[6] de Wit E , de Laat W . A decade of 3C technologies: insights into nuclear organization. Genes Dev. 2012;26(1):11‐24. 22215806 10.1101/gad.179804.111 PMC3258961

[7] Oluwadare O , Highsmith M , Cheng J . An overview of methods for reconstructing 3‐D chromosome and genome structures from hi‐C data. Biol Proced Online. 2019;21(1):7. 31049033 10.1186/s12575-019-0094-0 PMC6482566

[8] Sun X , Zhang J , Cao C . CTCF and its partners: shaper of 3D genome during development. Genes (Basel). 2022;13(8):1383. 36011294 10.3390/genes13081383 PMC9407368

[9] Gomez‐Diaz E , Corces VG . Architectural proteins: regulators of 3D genome organization in cell fate. Trends Cell Biol. 2014;24(11):703‐711. 25218583 10.1016/j.tcb.2014.08.003 PMC4254322

[10] Zheng H , Xie W . The role of 3D genome organization in development and cell differentiation. Nat Rev Mol Cell Biol. 2019;20(9):535‐550. 31197269 10.1038/s41580-019-0132-4

[11] Goldman JA , Poss KD . Gene regulatory programmes of tissue regeneration. Nat Rev Genet. 2020;21(9):511‐525. 32504079 10.1038/s41576-020-0239-7 PMC7448550

[12] Marchal C , Sima J , Gilbert DM . Control of DNA replication timing in the 3D genome. Nat Rev Mol Cell Biol. 2019;20(12):721‐737. 31477886 10.1038/s41580-019-0162-y PMC11567694

[13] Tanwar VS , Jose CC , Cuddapah S . Role of CTCF in DNA damage response. Mut Res Rev Mut Res. 2019;780:61‐68. 10.1016/j.mrrev.2018.02.002 PMC6690391 31395350

[14] Mohanta TK , Mishra AK , Al‐Harrasi A . The 3D genome: from structure to function. Int J Mol Sci. 2021;22(21):11585. 34769016 10.3390/ijms222111585 PMC8584255

[15] Cremer T , Cremer C , Baumann H , et al. Rabl's model of the interphase chromosome arrangement tested in Chinese hamster cells by premature chromosome condensation and laser‐UV‐microbeam experiments. Hum Genet. 1982;60(1):46‐56. 7076247 10.1007/BF00281263

[16] Tanabe H , Muller S , Neusser M , et al. Evolutionary conservation of chromosome territory arrangements in cell nuclei from higher primates. Proc Natl Acad Sci U S A. 2002;99(7):4424‐4429. 11930003 10.1073/pnas.072618599 PMC123664

[17] Bolzer A , Kreth G , Solovei I , et al. Three‐dimensional maps of all chromosomes in human male fibroblast nuclei and prometaphase rosettes. PLoS Biol. 2005;3(5):e157. 15839726 10.1371/journal.pbio.0030157 PMC1084335

[18] Rosin LF , Crocker O , Isenhart RL , Nguyen SC , Xu Z , Joyce EF . Chromosome territory formation attenuates the translocation potential of cells. Elife. 2019;8:e49553. 31682226 10.7554/eLife.49553 PMC6855801

[19] Lieberman‐Aiden E , van Berkum NL , Williams L , et al. Comprehensive mapping of long‐range interactions reveals folding principles of the human genome. Science. 2009;326(5950):289‐293. 19815776 10.1126/science.1181369 PMC2858594

[20] Nichols MH , Corces VG . Principles of 3D compartmentalization of the human genome. Cell Rep. 2021;35(13):109330. 34192544 10.1016/j.celrep.2021.109330 PMC8265014

[21] Solovei I , Thanisch K , Feodorova Y . How to rule the nucleus: divide et impera. Curr Opin Cell Biol. 2016;40:47‐59. 26938331 10.1016/j.ceb.2016.02.014

[22] van Steensel B , Belmont AS . Lamina‐associated domains: links with chromosome architecture, heterochromatin, and gene repression. Cell. 2017;169(5):780‐791. 28525751 10.1016/j.cell.2017.04.022 PMC5532494

[23] Wijchers PJ , Krijger PHL , Geeven G , et al. Cause and consequence of tethering a SubTAD to different nuclear compartments. Mol Cell. 2016;61(3):461‐473. 26833089 10.1016/j.molcel.2016.01.001 PMC4747903

[24] Wang SY , Su JH , Beliveau BJ , et al. Spatial organization of chromatin domains and compartments in single chromosomes. Science. 2016;353(6299):598‐602. 27445307 10.1126/science.aaf8084 PMC4991974

[25] Nora EP , Lajoie BR , Schulz EG , et al. Spatial partitioning of the regulatory landscape of the X‐inactivation Centre. Nature. 2012;485(7398):381‐385. 22495304 10.1038/nature11049 PMC3555144

[26] Dixon JR , Selvaraj S , Yue F , et al. Topological domains in mammalian genomes identified by analysis of chromatin interactions. Nature. 2012;485(7398):376‐380. 22495300 10.1038/nature11082 PMC3356448

[27] Phillips‐Cremins JE , Sauria ME , Sanyal A , et al. Architectural protein subclasses shape 3D organization of genomes during lineage commitment. Cell. 2013;153(6):1281‐1295. 23706625 10.1016/j.cell.2013.04.053 PMC3712340

[28] Beagan JA , Phillips‐Cremins JE . On the existence and functionality of topologically associating domains. Nat Genet. 2020;52(1):8‐16. 31925403 10.1038/s41588-019-0561-1 PMC7567612

[29] Gómez‐Marín C , Tena JJ , Acemel RD , et al. Evolutionary comparison reveals that diverging CTCF sites are signatures of ancestral topological associating domains borders. Proc Natl Acad Sci U S A. 2015;112(24):7542‐7547. 26034287 10.1073/pnas.1505463112 PMC4475986

[30] Dixon JR , Gorkin DU , Ren B . Chromatin domains: the unit of chromosome organization. Mol Cell. 2016;62(5):668‐680. 27259200 10.1016/j.molcel.2016.05.018 PMC5371509

[31] Lupianez DG , Kraft K , Heinrich V , et al. Disruptions of topological chromatin domains cause pathogenic rewiring of gene‐enhancer interactions. Cell. 2015;161(5):1012‐1025. 25959774 10.1016/j.cell.2015.04.004 PMC4791538

[32] Rao SS , Huntley MH , Durand NC , et al. A 3D map of the human genome at kilobase resolution reveals principles of chromatin looping. Cell. 2014;159(7):1665‐1680. 25497547 10.1016/j.cell.2014.11.021 PMC5635824

[33] Fudenberg G , Imakaev M , Lu C , Goloborodko A , Abdennur N , Mirny LA . Formation of chromosomal domains by loop extrusion. Cell Rep. 2016;15(9):2038‐2049. 27210764 10.1016/j.celrep.2016.04.085 PMC4889513

[34] Tang Z , Luo OJ , Li X , et al. CTCF‐mediated human 3D genome architecture reveals chromatin topology for transcription. Cell. 2015;163(7):1611‐1627. 26686651 10.1016/j.cell.2015.11.024 PMC4734140

[35] Kim Y , Shi Z , Zhang H , Finkelstein IJ , Yu H . Human cohesin compacts DNA by loop extrusion. Science. 2019;366(6471):1345‐1349. 31780627 10.1126/science.aaz4475 PMC7387118

[36] Hsieh TS , Cattoglio C , Slobodyanyuk E , et al. Resolving the 3D landscape of transcription‐linked mammalian chromatin folding. Mol Cell. 2020;78:539‐553.e8. 32213323 10.1016/j.molcel.2020.03.002 PMC7703524

[37] Deng W , Lee J , Wang H , et al. Controlling long‐range genomic interactions at a native locus by targeted tethering of a looping factor. Cell. 2012;149(6):1233‐1244. 22682246 10.1016/j.cell.2012.03.051 PMC3372860

[38] Li J , Huang K , Hu G , et al. An alternative CTCF isoform antagonizes canonical CTCF occupancy and changes chromatin architecture to promote apoptosis. Nat Commun. 2019;10(1):1535. 30948729 10.1038/s41467-019-08949-w PMC6449404

[39] Hu G , Dong X , Gong S , Song Y , Hutchins AP , Yao H . Systematic screening of CTCF binding partners identifies that BHLHE40 regulates CTCF genome‐wide distribution and long‐range chromatin interactions. Nucleic Acids Res. 2020;48(17):9606‐9620. 32885250 10.1093/nar/gkaa705 PMC7515718

[40] Weintraub AS , Li CH , Zamudio AV , et al. YY1 is a structural regulator of enhancer‐promoter loops. Cell. 2017;171(7):1573‐1588.e1528. 29224777 10.1016/j.cell.2017.11.008 PMC5785279

[41] Nora EP , Goloborodko A , Valton AL , et al. Targeted degradation of CTCF decouples local insulation of chromosome domains from genomic compartmentalization. Cell. 2017;169(5):930‐944.e922. 28525758 10.1016/j.cell.2017.05.004 PMC5538188

[42] Hsieh TS , Cattoglio C , Slobodyanyuk E , Hansen AS , Darzacq X , Tjian R . Enhancer‐promoter interactions and transcription are largely maintained upon acute loss of CTCF, cohesin, WAPL or YY1. Nat Genet. 2022;54(12):1919‐1932. 36471071 10.1038/s41588-022-01223-8 PMC9729117

[43] Dekker J , Rippe K , Dekker M , Kleckner N . Capturing chromosome conformation. Science. 2002;295(5558):1306‐1311. 11847345 10.1126/science.1067799

[44] Zhao Z , Tavoosidana G , Sjolinder M , et al. Circular chromosome conformation capture (4C) uncovers extensive networks of epigenetically regulated intra‐ and interchromosomal interactions. Nat Genet. 2006;38(11):1341‐1347. 17033624 10.1038/ng1891

[45] Simonis M , Klous P , Splinter E , et al. Nuclear organization of active and inactive chromatin domains uncovered by chromosome conformation capture‐on‐chip (4C). Nat Genet. 2006;38(11):1348‐1354. 17033623 10.1038/ng1896

[46] Dostie J , Richmond TA , Arnaout RA , et al. Chromosome conformation capture carbon copy (5C): a massively parallel solution for mapping interactions between genomic elements. Genome Res. 2006;16(10):1299‐1309. 16954542 10.1101/gr.5571506 PMC1581439

[47] Du Z , Zheng H , Huang B , et al. Allelic reprogramming of 3D chromatin architecture during early mammalian development. Nature. 2017;547(7662):232‐235. 28703188 10.1038/nature23263

[48] Liang Z , Li G , Wang Z , et al. BL‐Hi‐C is an efficient and sensitive approach for capturing structural and regulatory chromatin interactions. Nat Commun. 2017;8(1):1622. 29158486 10.1038/s41467-017-01754-3 PMC5696378

[49] Hsieh TH , Weiner A , Lajoie B , Dekker J , Friedman N , Rando OJ . Mapping nucleosome resolution chromosome folding in yeast by micro‐C. Cell. 2015;162(1):108‐119. 26119342 10.1016/j.cell.2015.05.048 PMC4509605

[50] Hua P , Badat M , Hanssen LLP , et al. Defining genome architecture at base‐pair resolution. Nature. 2021;595(7865):125‐129. 34108683 10.1038/s41586-021-03639-4

[51] Aljahani A , Hua P , Karpinska MA , Quililan K , Davies JOJ , Oudelaar AM . Analysis of sub‐kilobase chromatin topology reveals nano‐scale regulatory interactions with variable dependence on cohesin and CTCF. Nat Commun. 2022;13(1):2139. 35440598 10.1038/s41467-022-29696-5 PMC9019034

[52] Fullwood MJ , Liu MH , Pan YF , et al. An oestrogen‐receptor‐alpha‐bound human chromatin interactome. Nature. 2009;462(7269):58‐64. 19890323 10.1038/nature08497 PMC2774924

[53] Mumbach MR , Rubin AJ , Flynn RA , et al. HiChIP: efficient and sensitive analysis of protein‐directed genome architecture. Nat Methods. 2016;13(11):919‐922. 27643841 10.1038/nmeth.3999 PMC5501173

[54] Mifsud B , Tavares‐Cadete F , Young AN , et al. Mapping long‐range promoter contacts in human cells with high‐resolution capture hi‐C. Nat Genet. 2015;47(6):598‐606. 25938943 10.1038/ng.3286

[55] Chai H , Tjong H , Li P , et al. ChIATAC is an efficient strategy for multi‐omics mapping of 3D epigenomes from low‐cell inputs. Nat Commun. 2023;14(1):213. 36639381 10.1038/s41467-023-35879-5 PMC9839710

[56] Nagano T , Lubling Y , Stevens TJ , et al. Single‐cell hi‐C reveals cell‐to‐cell variability in chromosome structure. Nature. 2013;502(7469):59‐64. 24067610 10.1038/nature12593 PMC3869051

[57] Tan L , Xing D , Chang CH , Li H , Xie XS . Three‐dimensional genome structures of single diploid human cells. Science. 2018;361(6405):924‐928. 30166492 10.1126/science.aat5641 PMC6360088

[58] Li G , Liu Y , Zhang Y , et al. Joint profiling of DNA methylation and chromatin architecture in single cells. Nat Methods. 2019;16(10):991‐993. 31384045 10.1038/s41592-019-0502-z PMC6765429

[59] Beagrie RA , Scialdone A , Schueler M , et al. Complex multi‐enhancer contacts captured by genome architecture mapping. Nature. 2017;543(7646):519‐524. 28273065 10.1038/nature21411 PMC5366070

[60] Winick‐Ng W , Kukalev A , Harabula I , et al. Cell‐type specialization is encoded by specific chromatin topologies. Nature. 2021;599(7886):684‐691. 34789882 10.1038/s41586-021-04081-2 PMC8612935

[61] Quinodoz SA , Ollikainen N , Tabak B , et al. Higher‐order inter‐chromosomal hubs shape 3D genome Organization in the Nucleus. Cell. 2018;174(3):744‐757.e724. 29887377 10.1016/j.cell.2018.05.024 PMC6548320

[62] Zheng M , Tian SZ , Capurso D , et al. Multiplex chromatin interactions with single‐molecule precision. Nature. 2019;566(7745):558‐562. 30778195 10.1038/s41586-019-0949-1 PMC7001875

[63] Kempfer R , Pombo A . Methods for mapping 3D chromosome architecture. Nat Rev Genet. 2020;21(4):207‐226. 31848476 10.1038/s41576-019-0195-2

[64] Lai B , Tang Q , Jin W , et al. Trac‐looping measures genome structure and chromatin accessibility. Nat Methods. 2018;15(9):741‐747. 30150754 10.1038/s41592-018-0107-y PMC7212307

[65] Liu S , Cao Y , Cui K , Tang Q , Zhao K . Hi‐TrAC reveals division of labor of transcription factors in organizing chromatin loops. Nat Commun. 2022;13(1):6679. 36335136 10.1038/s41467-022-34276-8 PMC9637178

[66] Moris N , Pina C , Arias AM . Transition states and cell fate decisions in epigenetic landscapes. Nat Rev Genet. 2016;17(11):693‐703. 27616569 10.1038/nrg.2016.98

[67] Dixon JR , Jung I , Selvaraj S , et al. Chromatin architecture reorganization during stem cell differentiation. Nature. 2015;518(7539):331‐336. 25693564 10.1038/nature14222 PMC4515363

[68] Pekowska A , Klaus B , Xiang W , et al. Gain of CTCF‐anchored chromatin loops Marks the exit from naive pluripotency. Cell Syst. 2018;7(5):482‐495.e410. 30414923 10.1016/j.cels.2018.09.003 PMC6327227

[69] Beagan JA , Duong MT , Titus KR , et al. YY1 and CTCF orchestrate a 3D chromatin looping switch during early neural lineage commitment. Genome Res. 2017;27(7):1139‐1152. 28536180 10.1101/gr.215160.116 PMC5495066

[70] Jain S , Ba Z , Zhang Y , Dai HQ , Alt FW . CTCF‐binding elements mediate accessibility of RAG substrates during chromatin scanning. Cell. 2018;174(1):102‐116.e114. 29804837 10.1016/j.cell.2018.04.035 PMC6026039

[71] Olbrich T , Vega‐Sendino M , Tillo D , et al. CTCF is a barrier for 2C‐like reprogramming. Nat Commun. 2021;12(1):4856. 34381034 10.1038/s41467-021-25072-x PMC8358036

[72] Kubo N , Ishii H , Xiong X , et al. Promoter‐proximal CTCF binding promotes distal enhancer‐dependent gene activation. Nat Struct Mol Biol. 2021;28(2):152‐161. 33398174 10.1038/s41594-020-00539-5 PMC7913465

[73] Gong S , Hu G , Guo R , et al. CTCF acetylation at lysine 20 is required for the early cardiac mesoderm differentiation of embryonic stem cells. Cell Regen. 2022;11(1):34. 36117192 10.1186/s13619-022-00131-w PMC9482892

[74] Wei C , Jia L , Huang X , et al. CTCF organizes inter‐a compartment interactions through RYBP‐dependent phase separation. Cell Res. 2022;32(8):744‐760. 35768498 10.1038/s41422-022-00676-0 PMC9343660

[75] Wakayama T , Perry AC , Zuccotti M , Johnson KR , Yanagimachi R . Full‐term development of mice from enucleated oocytes injected with cumulus cell nuclei. Nature. 1998;394(6691):369‐374. 9690471 10.1038/28615

[76] Takahashi K , Yamanaka S . Induction of pluripotent stem cells from mouse embryonic and adult fibroblast cultures by defined factors. Cell. 2006;126(4):663‐676. 16904174 10.1016/j.cell.2006.07.024

[77] Hou P , Li Y , Zhang X , et al. Pluripotent stem cells induced from mouse somatic cells by small‐molecule compounds. Science. 2013;341(6146):651‐654. 23868920 10.1126/science.1239278

[78] Ping W , Hu J , Hu G , et al. Genome‐wide DNA methylation analysis reveals that mouse chemical iPSCs have closer epigenetic features to mESCs than OSKM‐integrated iPSCs. Cell Death Dis. 2018;9(2):187. 29416007 10.1038/s41419-017-0234-x PMC5833453

[79] Papp B , Plath K . Reprogramming to pluripotency: stepwise resetting of the epigenetic landscape. Cell Res. 2011;21(3):486‐501. 21321600 10.1038/cr.2011.28 PMC3193418

[80] Deng W , Jacobson EC , Collier AJ , Plath K . The transcription factor code in iPSC reprogramming. Curr Opin Genet Dev. 2021;70:89‐96. 34246082 10.1016/j.gde.2021.06.003 PMC9469655

[81] Chronis C , Fiziev P , Papp B , et al. Cooperative binding of transcription factors orchestrates reprogramming. Cell. 2017;168(3):442‐459.e420. 28111071 10.1016/j.cell.2016.12.016 PMC5302508

[82] Knaupp AS , Buckberry S , Pflueger J , et al. Transient and permanent reconfiguration of chromatin and transcription factor occupancy drive reprogramming. Cell Stem Cell. 2017;21(6):834‐845.e836. 29220667 10.1016/j.stem.2017.11.007

[83] Krijger Peter Hugo L , Di Stefano B , de Wit E , et al. Cell‐of‐origin‐specific 3D genome structure acquired during somatic cell reprogramming. Cell Stem Cell. 2016;18(5):597‐610. 26971819 10.1016/j.stem.2016.01.007 PMC4858530

[84] Stadhouders R , Vidal E , Serra F , et al. Transcription factors orchestrate dynamic interplay between genome topology and gene regulation during cell reprogramming. Nat Genet. 2018;50(2):238‐249. 29335546 10.1038/s41588-017-0030-7 PMC5810905

[85] Di Giammartino DC , Kloetgen A , Polyzos A , et al. KLF4 is involved in the organization and regulation of pluripotency‐associated three‐dimensional enhancer networks. Nat Cell Biol. 2019;21(10):1179‐1190. 31548608 10.1038/s41556-019-0390-6 PMC7339746

[86] Wang J , Yu H , Ma Q , et al. Phase separation of OCT4 controls TAD reorganization to promote cell fate transitions. Cell Stem Cell. 2021;28(10):1868‐1883.e1811. 34038708 10.1016/j.stem.2021.04.023

[87] Song Y , Liang Z , Zhang J , et al. CTCF functions as an insulator for somatic genes and a chromatin remodeler for pluripotency genes during reprogramming. Cell Rep. 2022;39(1):110626. 35385732 10.1016/j.celrep.2022.110626

[88] Alpsoy A , Sood S , Dykhuizen EC . At the crossroad of gene regulation and genome organization: potential roles for ATP‐dependent chromatin remodelers in the regulation of CTCF‐mediated 3D architecture. Biology. 2021;10(4):272. 33801596 10.3390/biology10040272 PMC8066914

[89] Li N , Kong M , Zeng S , et al. Brahma related gene 1 (Brg1) contributes to liver regeneration by epigenetically activating the Wnt/β‐catenin pathway in mice. FASEB J. 2019;33(1):327‐338. 30001167 10.1096/fj.201800197R

[90] Sun X , Chuang J‐C , Kanchwala M , et al. Suppression of the SWI/SNF component Arid1a promotes mammalian regeneration. Cell Stem Cell. 2016;18(4):456‐466. 27044474 10.1016/j.stem.2016.03.001 PMC4826298

[91] Shi J , Whyte WA , Zepeda‐Mendoza CJ , et al. Role of SWI/SNF in acute leukemia maintenance and enhancer‐mediated Myc regulation. Genes Dev. 2013;27(24):2648‐2662. 24285714 10.1101/gad.232710.113 PMC3877755

[92] Barutcu AR , Lajoie BR , Fritz AJ , et al. SMARCA4 regulates gene expression and higher‐order chromatin structure in proliferating mammary epithelial cells. Genome Res. 2016;26(9):1188‐1201. 27435934 10.1101/gr.201624.115 PMC5052043

[93] Wang AW , Wang YJ , Zahm AM , Morgan AR , Wangensteen KJ , Kaestner KH . The dynamic chromatin architecture of the regenerating liver. Cell Mol Gastroenterol Hepatol. 2020;9(1):121‐143. 31629814 10.1016/j.jcmgh.2019.09.006 PMC6909351

[94] Kraft K , Yost KE , Murphy SE , et al. Polycomb‐mediated genome architecture enables long‐range spreading of H3K27 methylation. Proc Natl Acad Sci U S A. 2022;119(22):e2201883119. 35617427 10.1073/pnas.2201883119 PMC9295753

[95] Papadogkonas G , Papamatheakis DA , Spilianakis C . 3D genome organization as an epigenetic determinant of transcription regulation in T cells. Front Immunol. 2022;13:921375. 35812421 10.3389/fimmu.2022.921375 PMC9257000

[96] Schuettengruber B , Bourbon H‐M , Di Croce L , Cavalli G . Genome regulation by Polycomb and Trithorax: 70 years and counting. Cell. 2017;171(1):34‐57. 28938122 10.1016/j.cell.2017.08.002

[97] Dupret B , Völkel P , Vennin C , Toillon RA , Le Bourhis X , Angrand PO . The histone lysine methyltransferase Ezh2 is required for maintenance of the intestine integrity and for caudal fin regeneration in zebrafish. Biochim Biophys Acta Gene Regul Mech. 2017;1860(10):1079‐1093. 28887218 10.1016/j.bbagrm.2017.08.011

[98] Ai S , Yu X , Li Y , et al. Divergent requirements for EZH1 in heart development versus regeneration. Circ Res. 2017;121(2):106‐112. 28512107 10.1161/CIRCRESAHA.117.311212 PMC5527745

[99] Li H , Lai P , Jia J , et al. RNA helicase DDX5 inhibits reprogramming to pluripotency by miRNA‐based repression of RYBP and its PRC1‐dependent and ‐independent functions. Cell Stem Cell. 2017;20(4):462‐477.e466. 28111200 10.1016/j.stem.2016.12.002

[100] Yao M , Zhou X , Zhou J , et al. PCGF5 is required for neural differentiation of embryonic stem cells. Nat Commun. 2018;9(1):1463. 29765032 10.1038/s41467-018-03781-0 PMC5954019

[101] Cohen I , Zhao D , Menon G , et al. PRC1 preserves epidermal tissue integrity independently of PRC2. Genes Dev. 2019;33(1–2):55‐60. 30567998 10.1101/gad.319939.118 PMC6317312

[102] Zhang C , Macchi F , Magnani E , Sadler KC . Chromatin states shaped by an epigenetic code confer regenerative potential to the mouse liver. Nat Commun. 2021;12(1):4110. 34226551 10.1038/s41467-021-24466-1 PMC8257577

[103] Cheutin T , Cavalli G . The multiscale effects of polycomb mechanisms on 3D chromatin folding. Crit Rev Biochem Mol Biol. 2019;54(5):399‐417. 31698957 10.1080/10409238.2019.1679082

[104] Murad R , Macias‐Muñoz A , Wong A , Ma X , Mortazavi A . Coordinated gene expression and chromatin regulation during hydra head regeneration. Genome Biol Evol. 2021;13(12):evab221. 34877597 10.1093/gbe/evab221 PMC8651858

[105] Vizcaya‐Molina E , Klein CC , Serras F , Mishra RK , Guigó R , Corominas M . Damage‐responsive elements in drosophila regeneration. Genome Res. 2018;28(12):1852‐1866. 30459214 10.1101/gr.233098.117 PMC6280756

[106] Kang J , Hu J , Karra R , et al. Modulation of tissue repair by regeneration enhancer elements. Nature. 2016;532(7598):201‐206. 27049946 10.1038/nature17644 PMC4844022

[107] Yan R , Cigliola V , Oonk KA , et al. An enhancer‐based gene‐therapy strategy for spatiotemporal control of cargoes during tissue repair. Cell Stem Cell. 2023;30:96‐111.e6. 36516837 10.1016/j.stem.2022.11.012 PMC9830588

[108] Dubois F , Sidiropoulos N , Weischenfeldt J , Beroukhim R . Structural variations in cancer and the 3D genome. Nat Rev Cancer. 2022;22(9):533‐546. 35764888 10.1038/s41568-022-00488-9 PMC10423586

[109] Ibrahim DM , Mundlos S . Three‐dimensional chromatin in disease: what holds us together and what drives us apart? Curr Opin Cell Biol. 2020;64:1‐9. 32036200 10.1016/j.ceb.2020.01.003

[110] Anania C , Lupianez DG . Order and disorder: abnormal 3D chromatin organization in human disease. Brief Funct Genomics. 2020;19(2):128‐138. 32025693 10.1093/bfgp/elz028 PMC7115703

[111] Wang H , Lou D , Wang Z . Crosstalk of genetic variants, allele‐specific DNA methylation, and environmental factors for complex disease risk. Front Genet. 2018;9:695. 30687383 10.3389/fgene.2018.00695 PMC6334214

[112] Anania C , Acemel RD , Jedamzick J , et al. In vivo dissection of a clustered‐CTCF domain boundary reveals developmental principles of regulatory insulation. Nat Genet. 2022;54(7):1026‐1036. 35817979 10.1038/s41588-022-01117-9 PMC9279147

[113] Weischenfeldt J , Dubash T , Drainas AP , et al. Pan‐cancer analysis of somatic copy‐number alterations implicates IRS4 and IGF2 in enhancer hijacking. Nat Genet. 2017;49(1):65‐74. 27869826 10.1038/ng.3722 PMC5791882

[114] Franke M , Ibrahim DM , Andrey G , et al. Formation of new chromatin domains determines pathogenicity of genomic duplications. Nature. 2016;538(7624):265‐269. 27706140 10.1038/nature19800

[115] Northcott PA , Lee C , Zichner T , et al. Enhancer hijacking activates GFI1 family oncogenes in medulloblastoma. Nature. 2014;511(7510):428‐434. 25043047 10.1038/nature13379 PMC4201514

[116] Gryder BE , Wachtel M , Chang K , et al. Miswired enhancer logic drives a cancer of the muscle lineage. iScience. 2020;23(5):101103. 32416589 10.1016/j.isci.2020.101103 PMC7226896

[117] Xu Z , Lee D‐S , Chandran S , et al. Structural variants drive context‐dependent oncogene activation in cancer. Nature. 2022;612(7940):564‐572. 36477537 10.1038/s41586-022-05504-4 PMC9810360

[118] Gregor A , Oti M , Kouwenhoven EN , et al. De novo mutations in the genome organizer CTCF cause intellectual disability. Am J Hum Genet. 2013;93(1):124‐131. 23746550 10.1016/j.ajhg.2013.05.007 PMC3710752

[119] Chen F , Yuan H , Wu W , et al. Three additional de novo CTCF mutations in Chinese patients help to define an emerging neurodevelopmental disorder. Am J Med Genet C Semin Med Genet. 2019;181(2):218‐225. 30893510 10.1002/ajmg.c.31698

[120] Konrad EDH , Nardini N , Caliebe A , et al. CTCF variants in 39 individuals with a variable neurodevelopmental disorder broaden the mutational and clinical spectrum. Genet Med. 2019;21(12):2723‐2733. 31239556 10.1038/s41436-019-0585-z PMC6892744

[121] Wang T , Hoekzema K , Vecchio D , et al. Large‐scale targeted sequencing identifies risk genes for neurodevelopmental disorders. Nat Commun. 2020;11(1):4932. 33004838 10.1038/s41467-020-18723-y PMC7530681

[122] Remeseiro S , Cuadrado A , Losada A . Cohesin in development and disease. Development. 2013;140(18):3715‐3718. 23981654 10.1242/dev.090605

[123] García‐Gutiérrez P , García‐Domínguez M . BETting on a transcriptional deficit as the Main cause for Cornelia de Lange syndrome. Front Mol Biosci. 2021;8:709232. 34386522 10.3389/fmolb.2021.709232 PMC8353280

[124] Panarotto M , Davidson IF , Litos G , Schleiffer A , Peters JM . Cornelia de Lange syndrome mutations in NIPBL can impair cohesin‐mediated DNA loop extrusion. Proc Natl Acad Sci U S A. 2022;119(18):e2201029119. 35476527 10.1073/pnas.2201029119 PMC9170158

[125] Olley G , Ansari M , Bengani H , et al. BRD4 interacts with NIPBL and BRD4 is mutated in a Cornelia de Lange‐like syndrome. Nat Genet. 2018;50(3):329‐332. 29379197 10.1038/s41588-018-0042-y PMC6469577

